# Peri-Personal Space Tracing by Hand-Blink Reflex Modulation in Patients with Chronic Disorders of Consciousness

**DOI:** 10.1038/s41598-020-58625-z

**Published:** 2020-02-03

**Authors:** Rocco Salvatore Calabrò, Antonino Chillura, Luana Billeri, Antonino Cannavò, Antonio Buda, Francesco Molonia, Alfredo Manuli, Placido Bramanti, Antonino Naro

**Affiliations:** IRCCS Centro Neurolesi Bonino Pulejo, Messina, Italy

**Keywords:** Central nervous system, Disorders of consciousness

## Abstract

The assessment of awareness in patients with chronic Disorders of Consciousness (DoC), including Unresponsive Wakefulness Syndrome (UWS) and Minimally Conscious State (MCS), is challenging. The level of awareness impairment may depend on the degree of deterioration of the large-scale cortical-thalamo-cortical networks induced by brain injury. Electrophysiological approaches may shed light on awareness presence in patients with DoC by estimating cortical functions related to the cortical-thalamo-cortical networks including, for example, the cortico-subcortical processes generating motor responses to the perturbation of the peri-personal space (PPS). We measured the amplitude, latency, and duration of the hand-blink reflex (HBR) responses by recording electromyography (EMG) signals from both the orbicularis oculi muscles while electrically stimulating the median nerve at the wrist. Such a BR is thought to be mediated by a neural circuit at the brainstem level. Despite its defensive-response nature, HBR can be modulated by the distance between the stimulated hand and the face. This suggests a functional top-down control of HBR as reflected by HBR features changes (latency, amplitude, and magnitude). We therefore estimated HBR responses in a sample of patients with DoC (8 MCS and 12 UWS, compared to 15 healthy controls −HC) while performing a motor task targeting the PPS. This consisted of passive movements in which the hand of the subject was positioned at different distances from the participant’s face. We aimed at demonstrating a residual top-down modulation of HBR properties, which could be useful to differentiate patients with DoC and, potentially, demonstrate awareness preservation. We found a decrease in latency, and an increase in duration and magnitude of HBR responses, which were all inversely related to the hand-to-face distance in HC and patients with MCS, but not in individuals with UWS. Our data suggest that only patients with MCS have preserved, residual, top-down modulation of the processes related to the PPS from higher-order cortical areas to sensory-motor integration network. Although the sample size was relatively small, being thus our data preliminary, HBR assessment seems a rapid, easy, and first-level tool to differentiate patients with MCS from those with UWS. We may also hypothesize that such a HBR modulation suggests awareness preservation.

## Introduction

The preservation of integration and differentiation processes of sensorimotor information within fronto-parietal regions is a critical element to consciousness generation and maintenance, according to the “neuronal complexity” and “integrated information” theories^1–7^.

The severe impairment of the neural pathways subtending these processes accounts for different disorders of consciousness (DoC), including Unresponsive Wakefulness Syndrome (UWS) and Minimally Conscious State (MCS). Awareness is totally lost in the former entity, whereas it is partially and variably impaired in the latter. Wakefulness is preserved in both such entities^8^. However, the misdiagnosis of patients with DoC, including those with covert awareness (i.e., patients with MCS diagnosed as UWS), is rather elevated^9–11^. In fact, about 32% of the behaviourally UWS patients can present signs of MCS, and about 69% of these patients might recover consciousness^12^. Of note, the highest misdiagnosis in this case is compared to the “clinical consensus” contrasted to the CSR-R^12^, as this scale is much more reliable and has contributed significantly to solving the high misdiagnosis rate. A possible reason of such misdiagnosis is that patients with UWS and MCS-minus may suffer from a cognitive-motor dissociation^13–15^. In such case, a patient with DoC is aware even though he/she cannot properly react to visual stimuli, follow commands, move limbs purposefully, and ultimately react to pain; such a patient can indeed respond to stimuli with increased or feeble, generalized, stereotyped, even delayed, gross body movements^6,7^. The origin of the pitfalls to confuse these patients can be akinetic mutism, cranial nerve palsy, critical care illness, awareness fluctuation, sensory impairment, thalamo-cortical deterioration degree, and pure motor-output failure^13–17^.

Another main problem when facing DoC diagnosis is the current lack of a gold standard toward DoC differential diagnosis^12^. Meanwhile, employing advanced functional neuroimaging and neurophysiological approaches can further reduce the misdiagnosis rate. Indeed, the misdiagnosis compared to the Coma Recovery Scale-Revised (CRS-R) is 11% if FDG-PET is used, and 4% if a mental imagery task with fMRI is adopted^18–21^. Therefore, there is great interest in identifying objective markers of awareness in patients with DoC.

Interestingly, the experimental measurement of sensorimotor integration and differentiation processes (e.g., using transcranial magnetic stimulation coupled with high-density EEG) has been shown to be promising in quantifying residual awareness regardless of overt behaviour^22^. Indeed, these approaches can explore the variety of sensorimotor integration processes occurring along the multiple, complex cortico-thalamo-cortical networks that altogether support behavioural output and awareness generation and maintenance^23^, regardless of the translation of such network activities in appreciable behaviours^22^. This experimental assessment can be thus used to corroborate (or not) the clinical diagnosis.

The way these networks, and the subtending processes, are activated differs whether sensory inputs come from the extra-personal or peri-personal space (PPS)^24–29^. The former is defined as the space beyond the arm reaching distance^30,31^. The latter refers to “the nearby representational space in terms of what was reachable - that is, within range of the arm’s reach”^32–34^. The multisensory information coming from the PPS are processed together to build a spatio-temporal reconstruction of the neighbouring environment in a body part-centred frame^35,36^. This internal reconstruction of PPS allows the building up of purposeful motor behaviours aimed at interacting with objects and persons (e.g., to grasp food and useful objects) and avoiding threats near the body (e.g., to avoid a bee flying towards the face)^37–40^. Thus, the PPS has two main functions: (i) behavioural, that is, to take advantage of opportunities within the own space (e.g., to grasp food and useful objects); and (ii) defensive, that is, to protect the body from potential threats occurring within this space (for example to avoid a bee flying towards the face)^38^. The internal reconstruction of PPS takes place within a vast fronto-parietal cortico-thalamo-cortical network encompassing putamen, parietal and frontal areas^39,41–44^ through which either purposeful movements are planned and executed (with particular regard to putamen, parietal, and frontal areas)^24,45–47^ or reflex responses related to PPS entrainment are regulated (top-down control) (with particular regard to the poly-sensory zone in the precentral gyrus and the ventral intraparietal area)^37,48–53^.

Even though extra-personal and PPS networks largely overlap, they can work in parallel, i.e., independently of each other^54^. This occurs in keeping with the double nature of PPS, i.e., defensive and behavioural^55^. Therefore, a functional dissociation between these networks can be hypothesized in the DoC population. This is in keeping with a possible preferential allocation of the post-injury available cognitive resources to control the subcortical areas that mediate the motor output, which are aimed at fostering defensive, reflexive behavioural responses^56^. In this way, a patient with DoC can be behaviourally unresponsive but covertly aware^57–62^. Thus, a patient with DoC may be unresponsive to the stimuli that fall within the extra-personal space when provided with the behavioural assessment (including the CRS-R), while being aware of the stimuli that specifically trigger the PPS, despite a behavioural responsiveness that is limited to reflexive behaviours^63–65^.

This demonstrates that PPS functional preservation may be useful for corroborating the clinical diagnoses of patients with DoC, regardless of behavioural unawareness. Furthermore, awareness preservation can be hypothesized in relation to the PPS. Such awareness stems from the sensorimotor information processing within the PPS itself. In fact, these networks largely overlap with those that are putatively involved in awareness generation and maintenance^7,54^. Thus, the activation of PPS includes different awareness levels depending on the features of the sensorimotor processes pertaining the PPS. These features include attention (top-down control), the location of a target on the PPS, the body part exposed to a threat, the cognitive and sensorimotor consequences of a stimulus (i.e., protective and goal-directed responses), the spatio-temporal correlations between two distinct stimuli approaching the PPS, the spatio-temporal properties of the PPS (e.g., effect of sight limitation on the magnitude of behavioural or brain signal responses), the social content of PPS, and the ongoing activity within the neural pathways (cortical, subcortical, brainstem, and spinal) mediating reflex responses triggered in the PPS^32,38,45,48,49,51,66,67^.

Awareness of the PPS may be tentatively demonstrated by measuring the top-down modulation of PPS-related behavioural responses. Top-down modulation of sensorimotor processes is a critical function of cognition, such as informing lower-order sensory systems of the ongoing sensorimotor scenario by conveying motoric planning to these systems^68^. The fact that such a top-down modulation could be a reflection of conscious awareness is controversial, as most top-down modulations are below the level of consciousness^69^. Therefore, a detrimental HBR modulation could simply reflect greater damage to the central sensorimotor network rather than an implication for consciousness. Nonetheless, the top-down modulation of sensorimotor processes related to the PPS responses may have some implications for consciousness when targets approach the PPS^70^. In fact, high-level top-down control occurs with new, potentially threatening, or complex tasks^61–63^. In other words, only the low-level, top-down modulation of sensorimotor processes can be unrelated to awareness, but not the high-level, top-down modulation processes, as in the case of PPS that would physiologically imply a high-level, top-down control processing^35,71–73^. This could also concern the patients with DoC, who show several adaptive plasticity changes across sensorimotor regions in the attempt to regain awareness^74–76^.

A suitable way to assess the top-down modulation of the behavioural responsiveness related to PPS is represented by the measurement of the hand blink reflex (HBR) responses. In such an assessment, it is possible to measure the amplitude, latency, and duration of the HBR responses by recording electromyography (EMG) signals from both the orbicularis oculi muscles while electrically stimulating the median nerve at the wrist. Such a BR is thought to be mediated by a neural circuit at the brainstem level^43^. Despite its defensive response nature, HBR can be modulated by the distance between the stimulated hand and the face, which implies an extensive, continuous mapping of the approaching targets within the PPS^38,48,49^. Thus, HBR modulation may reflect specific, top-down control processes within the cortico-thalamo-cortical networks supporting the PPS internal reconstruction and building up the behavioral responses^55^. This model is in keeping with either the “Swiss army-knife model” (all possible PPS-related behavioral responses in one cortico-thalamo-cortical map) or the “Specialist model” (as many cortico-thalamo-cortical maps as there are multiple the PPS-related behavioral responses, i.e., hand-, head-, and trunk-centered)^11–14^. Thus, demonstrating the preservation of PPS functions, by proving the conservation of HBR feature modulation with particular regard to HBR magnitude, might allow DoC differential diagnosis and, potentially, suggest awareness preservation, despite unawareness at the behavioural assessment.

## Materials and Methods

### Subjects

Twenty right-handed patients with DoC (eight MCS and twelve UWS) attending our Severe Acquired Brain Injury Unit were consecutively enrolled in this study over a two-year period. DoC condition was due to vascular, hypoxic-ischemic, or traumatic brain damage. We recruited 15 healthy, right-handed, age- and gender-matched individuals as a control group (HC). Demographic and clinical characteristics are reported in Table 1. Patients had to meet the criteria for vegetative state/UWS and MCS diagnosis^77^ to enter the study. Exclusion criteria were: absence of blink-reflex or facial nerve damage; absence of visual evoked potentials (elicited with goggles); administration of modifying cortical-excitability drugs other than L-Dopa, baclofen, and anti-epileptic drugs; critical conditions, such as inability to breathe independently, and hemodynamic instability; evidence of large brainstem damage at magnetic resonance imaging; pre-existing severe neurological or systemic diseases; severe impairment of the peripheral nerves (assessed by electromyography) and of somatosensory and motor evoked potentials from upper limbs (to rule out damage to neural pathways conflicting with our study purposes, i.e., implication for the impairment of consciousness); and severe spasticity to the upper limb. The present study was approved by the Ethics Committee of the IRCCS Centro Neurolesi Bonino Pulejo (Messina, Italy). Both HC and the legal guardian of each patient provided their written informed consent.

**Table 1:** Disorder of Consciousness (DoC) clinical-demographic characteristics.

DoC	Etiology	Gender	Age (y)	BI onset (m)	MRI	CRS-R
MCS+	T	M	70	12	FP_h	18
MCS+	T	F	48	11	FP_h	18
MCS+	V	M	39	20	SAH	15
MCS−	T	F	42	15	F_h	9
MCS−	T	M	39	11	FP_h	18
MCS+	T	F	67	16	multiple_h	18
MCS+	V	M	45	11	FTP_IS	11
MCS−	V	M	67	4	FP_h	12
Mean ± SD	5 T, 3 V	5 M, 3 F	52 ± 14	13 ± 5		15 ± 4
UWS	A	F	61	3	WMH	5
UWS	T	M	36	6	DAI + FP_h	5
UWS	T	M	47	2	multiple_h	4
UWS	V	F	60	3	FTP_IS	6
UWS	V	F	68	21	TP_IS	5
UWS	A	F	36	12	WMH	7
UWS	T	M	62	15	FP_h	4
UWS	A	F	42	8	WMH	7
UWS	T	M	64	21	DAI + TP_h	5
UWS	T	F	46	6	DAI + Fb_h	5
UWS	V	F	37	13	SAH	5
UWS	T	M	46	7	multiple_h	7
Mean ± SD	3 A, 6 T,3 V	7 M, 5 F	50 ± 12	10 ± 7		5 ± 1
(*)	0.3	0.4	0.8	0.3	0.2	<0.001

Abbreviations: CRS-R Coma Recovery Scale–Revised; BI brain injury; F female; M male; V vascular; T traumatic; A anoxic; MCS Minimally Conscious State; UWS Unresponsive Wakefulness Syndrome; MRI magnetic resonance imaging pattern (FP frontoparietal, _h hemorrhage, SAH subarachnoid hemorrhage, F frontal, FP fronto-parietal, Fb frontobasal, WMH white matter hyperintensity, DAI diffuse axonal injury, FTP fronto-temporo-parietal, TP temporo-parietal, _IS ischemia); (*) p-value of between group *t*-test at entry time.

### Experimental protocol

**Figure 1:**
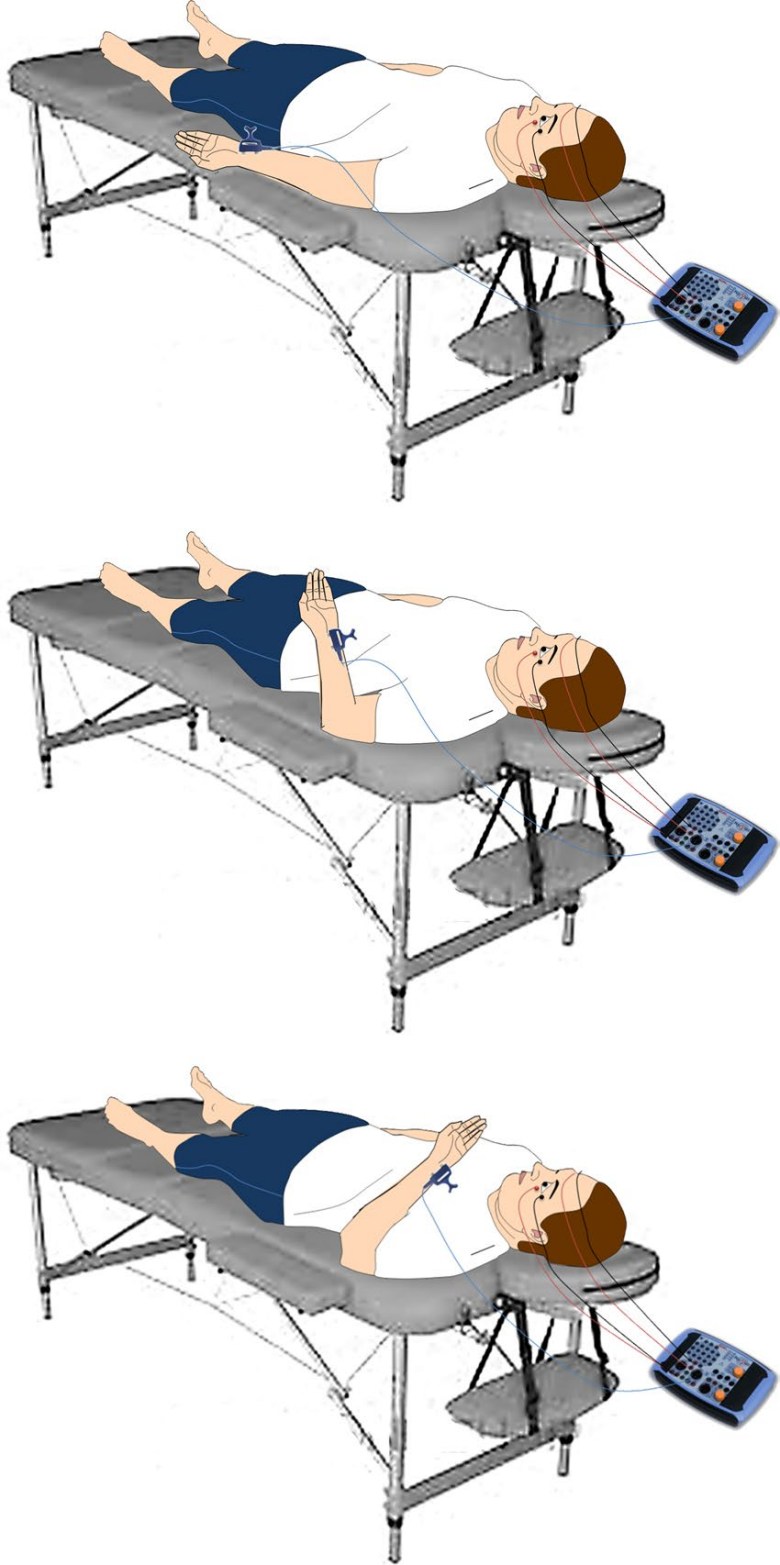
Schematic representation of the stimulation and recording setup.

First, patients were clinically evaluated for a month using the CRS-R to steadily define the level of behavioural responsiveness. Specifically, patients were assessed with the CRS-R at least five-to-six times within a 10-day period. The highest behavioral score obtained after these evaluations was then considered as a reference for diagnosis and follow-up^78^. Then, we measured the HBR features across different positions of the stimulated hand while progressively going toward and away from the face. The participant was lying supine on his/her bed in a semi-darkened environment, wearing ear tips, and with the eyes open (this was guaranteed by CRS-R arousal protocol in patients with DoC). The upper limb to be stimulated was put prone along the trunk to move the forearm towards the face, without touching it (Fig. 1). A headrest was used to hold the head in place and to minimize head movements. We first obtained a well-defined and stable HBR, by increasing the stimulus intensity until a clear HBR was observed in three consecutive trials, at a maximal intensity described as tolerable by all the HC^79^. Then, HBR was recorded putting the forearm extended on the arm (ultrafar position = 180 deg), the forearm flexed at 90 deg on the arm (far position), and the forearm flexed on the arm at 10 deg (near position)^80,81^. Consequently, the hand was visible to the subject only in the far and near positions. The other upper limb was held along the body throughout the experiment. We delivered 30 electric stimuli to the right and left wrist in two separate blocks, each of which consisted of 10 stimuli delivered in the ultrafar, far, and near position; the order of blocks was random but balanced across participants. Eyes-open condition and vigilance were guaranteed by applying the CRS-R arousal protocol, when necessary.

We stimulated the median nerve at the wrist through square-wave pulses delivered by using a bipolar electrode with the cathode proximally and a pulse width of 500 μs (Digitimer D-160 stimulator; Digitimer Ltd, Welwyn Garden City, Herts, UK). Electric shocks were delivered randomly (with an interstimulus interval of at least 10 s). EMG was recorded with Ag-AgCl surface electrodes placed on the orbicularis oculi muscles (one electrode over the mid-lower eyelid, the other few centimetres laterally to the outer canthus). EMG signals were amplified and bandpass filtered (32–1000 Hz) by a Digitimer D-150 amplifier and stored at a sampling rate of 10 kHz on a personal computer for offline analysis (Signal Software; Cambridge Electronic Design, Cambridge, UK). Then, given that there were no differences when stimulating right or left wrist, signals were full-wave rectified and averaged at the ipsilateral or controlateral recording sides on the left and right wrists (i.e., 60 responses for each recording side). The definition of HBR features was based on a visual inspection of the EMG recording that has been carried out in previous works^48,49,79^. Thereafter, we considered the bursts of EMG activity with an amplitude of at least 50 µV and a duration of at least 10 ms at a latency that was compatible with a reflex response (i.e., earlier than a voluntary reaction) for data analysis. We set the onsets and ends of the HBR responses at points where the mean of the samples within a fixed time window surpassed the baseline level by 2.5 standard deviations^82,83^. The amplitude was measured at the highest peak of the EMG burst; the area was calculated by multiplying the peak amplitude by the duration of the response.

### Statistical analysis

The data showed a normal distribution (Kolmogorov–Smirnov test p > 0.2). Measure equivalence at baseline between DoC groups was evaluated with a *t–*test. An ANOVA with the factors *hand-position* (three levels: near, far, and ultrafar), *recording-side* (two levels: ipsilateral and controlateral), and *group* (three levels: HC, MCS, and UWS) was used to investigate each HBR parameter (onset latency, duration, and magnitude, as measured by the area under the curve -AUC). Statistical significance was set at p < 0.05. *Post–hoc t–*tests were Bonferroni corrected.

Correlation between clinical (CRS-R) and electrophysiological measures (onset, duration, and AUC of HBR response) were tested using the logistic regression. We assumed that if there is a relationship between the categorical and continuous variable (i.e., a success/failure in the correspondence between the diagnosis based on the interpretation of the CRS-R scores and the HBR feature modulation by hand position thresholded at a percentage deduced by HC data), it is thus possible to construct an accurate predictor of the diagnosis based on the interpretation of the CRS-R scores (categorical variable) from the HBR modulation (continuous variable). It can be concluded that two variables share a relationship and are indeed correlated whether the resulting classifier has a high degree of fit, is accurate, sensitive, and specific. Differences and correlations were considered significant at *p* < 0.05. Last, we sought out the sensibility/specificity of the test in differentiating MCS from UWS at the individual level by using the likelihood ratio (LR), which provides the probability that patients have such a disease or not by using a test^84^.

### Informed consent

Informed consent was obtained from all individual participants included in the study.

### Ethical approval

All procedures performed in studies involving human participants were in accordance with the ethical standards of the institutional and/or national research committee and with the 1964 Helsinki declaration and its later amendments or comparable ethical standards. The Ethics Committee of the IRCCS Centro Neurolesi Bonino Pulejo (Messina, Italy) approved the present study (ID: 32/2017). All participants gave their written informed consent.

## Results

**Figure 2:**
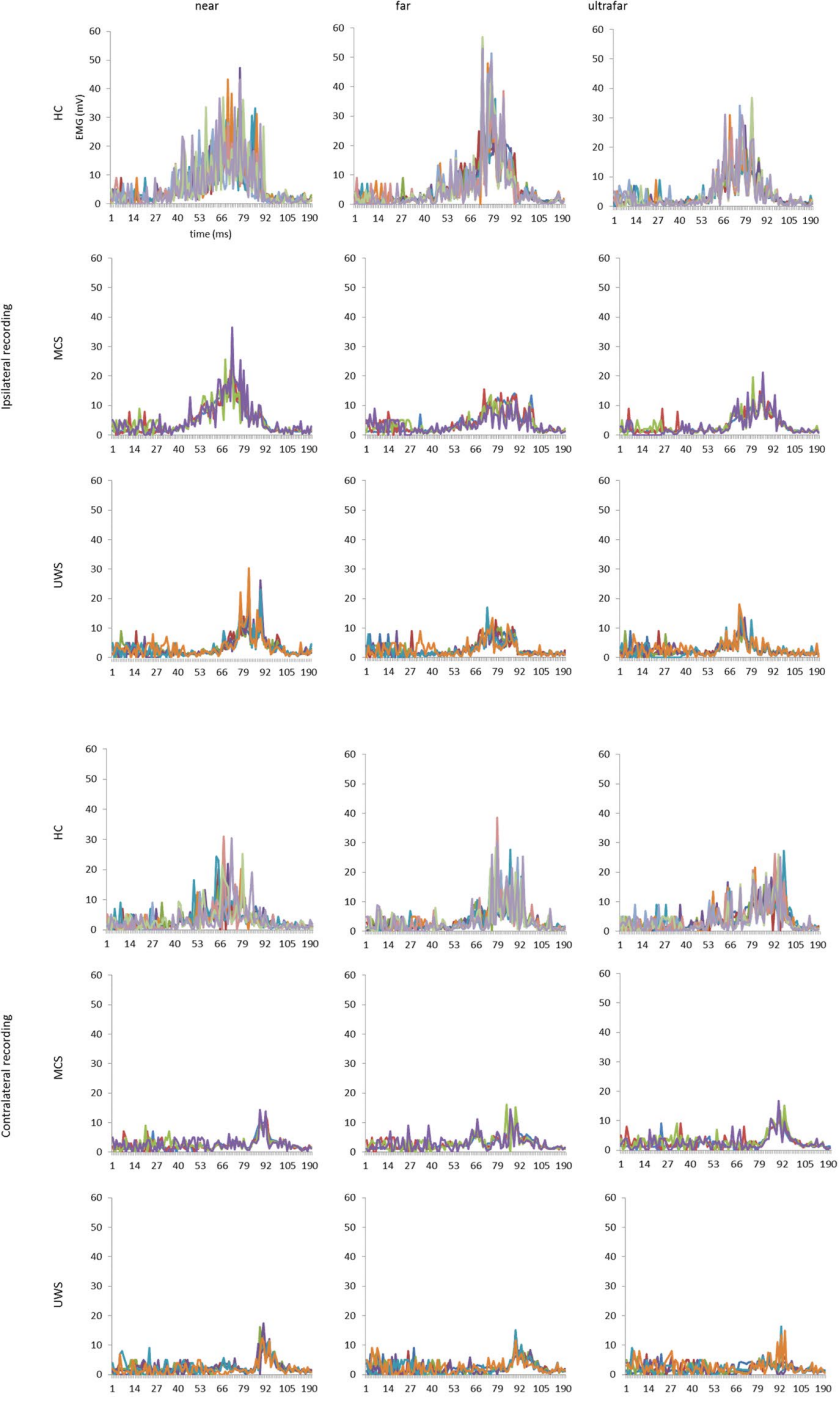
Rectified and superimposed average of each participant for each hand-position, group, and recording-side, and group-average HBR waveforms for each hand-position, and recording-side. Each participant is represented by a different colour. x-axis, time (ms); y-axis, EMG activity (mV).

**Figure 3:**
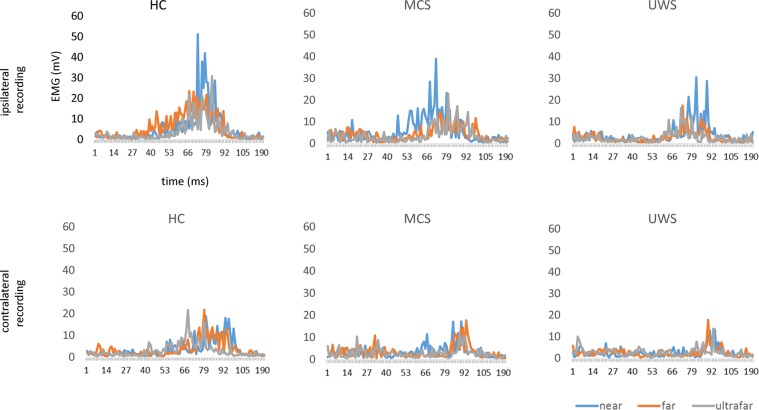
Rectified and superimposed group-average HBR for each hand-position, and recording-side. Each hand position is represented by a different color. x-axis, time (ms); y-axis, EMG activity (mV).

All the individuals completed the experimental procedure, without any adverse effect. Eleven out of 15 HC individuals showed HBR, whereas all the patients with DoC showed a repeatable HBR. Figures 2 and 3 illustrate the individual and group-average HBR responses, respectively, for each hand position and recording side. We first sought the differences between the groups in the overall changes of HBR features (latency, duration, and magnitude –AUC) (*group* × *hand-position* × *recording-side* in Table 2). Then we estimated the effects of each hand-position and recording side on each HBR feature in each group (*hand-position* × *recording-side* and *post-hoc* tests in Table 2). Depending on a significant hand position effect, we sought the differences between the groups concerning hand-position and recording-side (Table 2).

**Table 2:** Statistical data of between and within group (g) effects (HC healthy controls, MCS Minimally Conscious State, UWS Unresponsive Wakefulness Syndrome) of the different hand positions (hp) and recording sides (rs) (*d* Cohen’s effect size) on hand blink reflex response latency, duration, and area under the curve (AUC).

*g* × *hp* × *rs*	*hp* × *rs*		*post-hoc* tests			*hp* group comparison			*rs* group comparison
**Latency**									
F_(4,128)_ = 3.2 p = 0.01 *d* = 0.62	HC	F_(2,28)_ = 6.1 p = 0.005 *d* = 0.88	hp rs	(all) p = 0.003 p < 0.001		MCS-UWS HC-UWS HC-MCS	(all) p = 0.3 (all) p < 0.0001 (all) p < 0.0001		(all) p = 0.3 (all) p = 0.3 (all) p = 0.3
	MCS	p = 0.3							
	UWS	p = 0.4							
**Duration**									
F_(4,128)_ = 5.6 p = 0.0003 *d* = 0.83	HC	F_(2,28)_ = 39 p < 0.0001 *d* = 2.24	hp rs	(all) p < 0.0001 p < 0.001		MCS-UWS HC-UWS HC-MCS	(all) p = 0.1 (all) p < 0.0001 (all) p < 0.0001		(all) p = 0.3 (all) p = 0.3 (all) p = 0.3
	MCS	F_(2,14)_ = 35 p < 0.0001 *d* = 2.12	hp rs	(all) p < 0.0001 p < 0.001					
	UWS	p = 0.4							
**AUC**									
F_(4,128)_ = 4.7 p = 0.001 *d* = 0.76	HC	F_(2,28)_ = 6.2 p = 0.004 *d* = 0.89	hp	far-near far-ultrafar near-ultrafar	p < 0.0001 p = 0.04 p = 0.02	MCS-UWS	(all) p < 0.001		(all) p < 0.0001
			rs	p < 0.001		HC-UWS	(all) p < 0.001		(all) p < 0.0001
	MCS	F_(2,14)_ = 3.5 p = 0.04 *d* = 0.67	hp	far-near far-ultrafar near-ultrafar	p = 0.04 p = 0.7 p = 0.02				
						HC-MCS	far-near	p < 0.001	(all) p < 0.0001
			rs	p < 0.001			far-ultrafar	p < 0.001	
	UWS	p = 0.4					near-ultrafar	p = 0.001	

**Figure 4:**
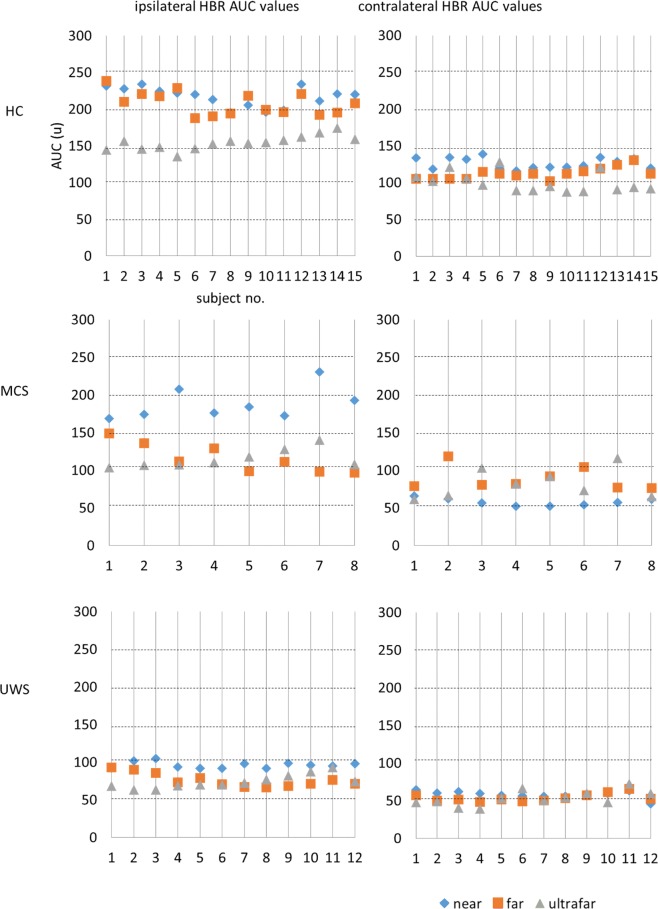
Individual values of the area under the curve (AUC) of the HBR response elicited for each hand-position (near, far, and ultrafar) and recording-side (ipsilateral and contralateral), in each group (HC, MCS, and UWS). Participant are reported on the x-axis, AUC (arbitrary units) on the y-axis.

The latency of the HBR response varied depending on the hand position (the nearer the hand, the shorter the latency) and the recording side (shorter in the ipsilateral side) in a different way among the groups (Table 2; Fig. 4). Specifically, the modulation of HBR latency was significant in HC but not in patients with MCS and UWS. By comparing the groups (Table 2), no difference in terms of latency modulation of HBR induced by the hand positions emerged between patients with MCS and UWS, whereas significant differences were found between HC and UWS, and HC and MCS.

The duration of the HBR response changed according to the hand position (the nearer the hand, the longer the duration) and the recording side (longer in the ipsilateral side) in a different way among the groups (Table 2; Fig. 4). Specifically, the modulation of HBR duration was significant in HC and in patients with MCS, but not in individuals with UWS (Table 2). By comparing the groups (Table 2), no difference in terms of duration modulation of HBR induced by the hand positions emerged between HC and patients with MCS, whereas significant differences were found between HC and UWS, and MCS and UWS.

The changing of the AUC of the HBR response depended on the hand position (the nearer the hand, the greater the AUC) and the recording side (greater in the ipsilateral side) in a different way among the groups (Table 2; Fig. 4). Specifically, the modulation of AUC was significant in HC and in patients with MCS, but not in individuals with UWS (Table 2). By comparing the groups (Table 2), a significant difference in terms of AUC modulation induced by the hand positions emerged between HC and UWS, HC and MCS, and MCS and UWS.

**Figure 5:**
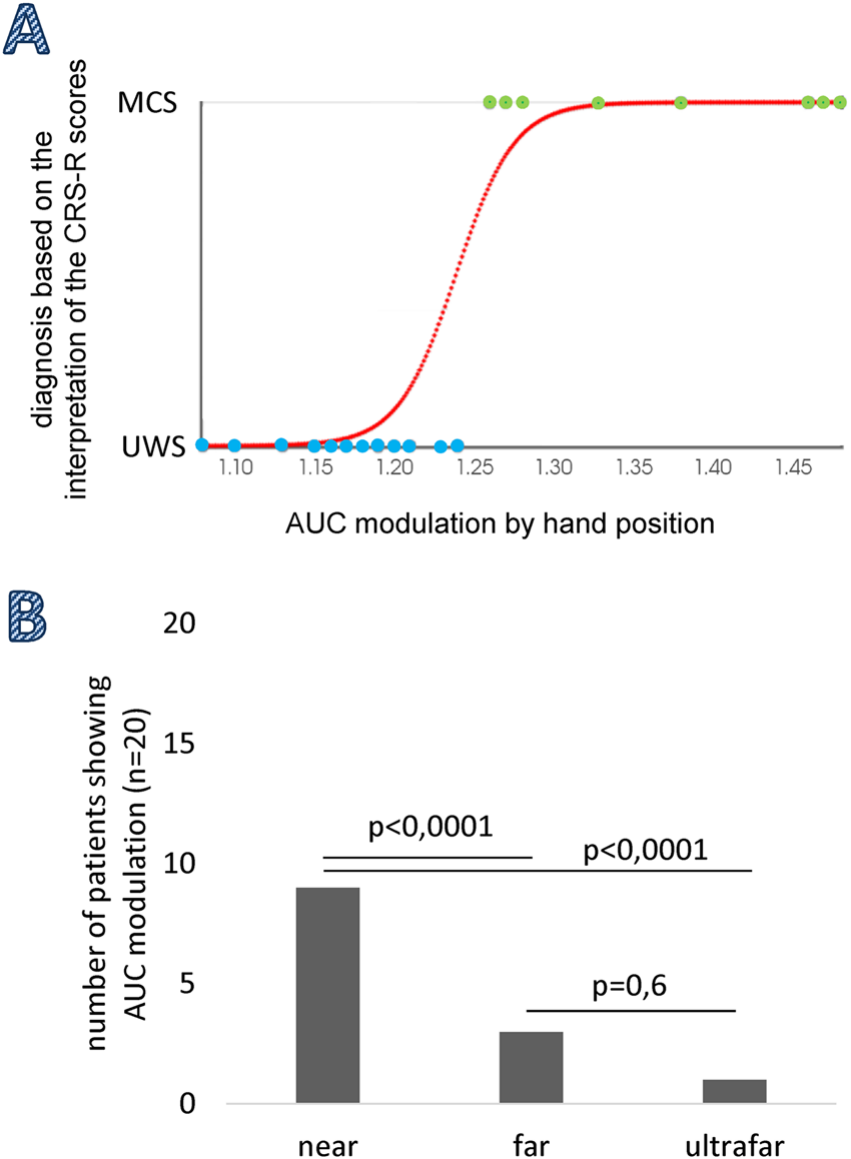
Panel A. Plot of the model (red curve) and data (blue dots patients with UWS, green dots patients with MCS) of the binomial success/failure of the correspondence between the diagnosis based on the interpretation of the CRS-R scores and the AUC modulation by hand position (thresholded at 126% as per AUC modulation in HCs). Panel B. Number of patients with DoC showing an AUC modulation (i.e., near > far > ultrafar) as a function of the hand position.

When calculating the clinical-electrophysiological correlation, the logistic regression of the binomial success/failure in the correspondence between the diagnosis based on the interpretation of the CRS-R scores and AUC modulation by hand position (thresholded at 126% as per AUC modulation in HCs) returned a χ^2^_(1,18)_ = 17, p < 0.0001 (Fig. 5A). When doing the local comparisons between the three hand positions, most of the patients showed an AUC modulation in the nearest hand position as compared to the other hand positions (p < 0.001), while the difference between the number of patients showing an AUC modulation in the far and ultrafar positions was not significant (Fig. 5B).

**Figure 6:**
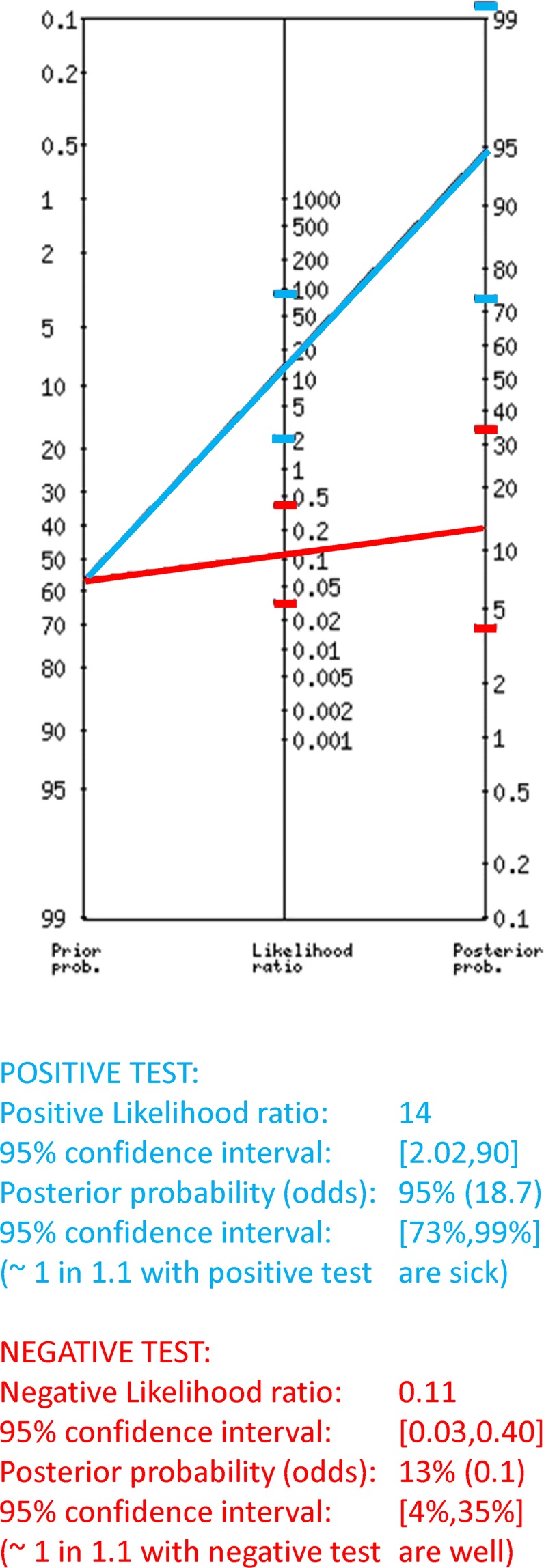
Fagan’s nomogram of the positive and negative LR of the AUC in the near position and ipsilateral recording toward the diagnosis based on the interpretation of the CRS-R scores.

Last, LR analysis revealed that the AUC modulation in the near position recorded ipsilaterally was very useful to point to DoC diagnosis at the individual level (Fig. 6). In fact, we found a LR for a positive result, (sensitivity/(1-specificity)) > 10, which indicates that the test result has a significant effect on increasing the probability of disease, and a LR for a negative result, ((1-sensitivity)/specificity) between 0.1 and 0.5, which indicates that the test has a moderate effect on decreasing probability of disease.

## Discussion

To the best of our knowledge, only one work investigated PPS in patients with DoC^70^. In this study, the authors assessed the EEG changes in response to near and far simple or multisensory stimuli, unravelling different brain responses according to DoC or cognitive-motor dissociation diagnosis. While there is an overlap between the study concept, the experimental setup and the observed measurements differ significantly. Thus, our study is to be considered a novelty.

Our results in the HC sample confirm that the HBR features (onset, duration, and AUC) are influenced by the distance of the stimulated hand from the face. In fact, HBR in the HC was of shorter latency, longer duration, and greater AUC when the hand was near than far the face. These findings are in keeping with the formerly reported tonic top-down modulation of HBR^48,49^, that is, the HBR excitability is selectively increased through the facilitation of specific brainstem circuits, which are pre-activated by part of given cortical networks allocated, but not exclusively, to PPS information processing. The increased responsiveness of the brainstem circuits (in particular, the HBR reticular interneurons) would facilitate the transmission of the signal to the facial motorneurons, resulting in an HBR of shorter latency and larger magnitude when approaching PPS^37,48,49^. The underlying network at supraspinal level includes different fronto-parietal areas that are responsible for somatotopically localizing the sensory stimuli (e.g., cutaneous, visual, or auditory)^85–87^, and then remapping them into an external, bodily-centered, motoric representation^88–93^. In other words, the brain forecasts the possible next position of a target within the PPS, depending on the currently available sensorimotor information. Therefore, the PPS network pre-activates cranial and spinal muscles, i.e., facilitates HBR magnitude, to predispose the body to the most appropriate motor response, depending on the proximity and the nature of a stimulus approaching the PPS. Consequently, the HBR responses will be of shorter latency, longer duration, and greater magnitude when the stimulus approaches the PPS rather than it goes away from. Such modulation may be in keeping with the necessity to have an as large as possible “safety margin” to be advantageous for survival^48,49,51,94–96^.

Our data suggest the preservation of both the PPS network and its top-down modulation in patients with MCS but not in those with UWS. In fact, the former showed a residual HBR feature modulation by hand-to-face distance. Moreover, the HBR modulation assessment demonstrated to be capable of differentiating patients with MCS from those with UWS, also at the individual level as indicated by the LR data.

Even though such a modulation does not necessarily reach the aware level (as the most of top-down modulations are below the level of consciousness), the preservation of the cortico-thalamo-cortical networks supporting such modulations is a fundamental prerequisite for the emergence of awareness^86^. Therefore, we can hypothesize that the magnitude of HBR modulation might reflect the degree of preservation of cortico-thalamo-cortical connectivity and, potentially, awareness. Moreover, patients with DoC show several adaptive plasticity changes across sensorimotor regions in the attempt to regain awareness^74–76^. Therefore, it might be more likely that inputs, including those that pertain to PPS, can reach the aware level to facilitate the recovery of awareness^74–76^. Notably, the over-strengthening of some physiological responses in extreme life conditions, like DoC represents, is not surprising as it has been demonstrated regarding other domains, e.g., pain perception^97^. This is in keeping with the higher AUC and duration of HBR in patients with MCS rather than in HC. Instead, patients with UWS did not show any significant modulation of the HBR features. Even though a systematic difference between near and ultrafar positions was appreciable, this difference failed to be significant when considering the whole pattern of AUC modulation within UWS and between the DoC groups.

This correlates with severe impairment of the cortico(-thalamo)-brainstem output, thus suggesting unawareness^98,99^. In other words, the patients with UWS do not seem to perceive and interpret the nature of the incoming stimulus (e.g., a threat), being thus able to show only non-purposeful, reflexive behavioral responses. This issue is also supported by the loss of HBR grading independent of either the hand position (i.e., possibility to see the hand) or the recording site. In this regard, the HBR magnitude was greater in the ipsilateral than the contralateral side, as physiologically occurs^37,48,49^. Conversely, the AUC modulation by part of hand position across sides was lost completely in the patients with UWS and partially in those with MCS. Indeed, HBR effects rely also on proprioceptive information about the stimulus location with respect to the face, as shown in the HC^37,48,49^ and the individuals with MCS. Such property was lost in the patients with UWS, as they had a tonic HBR. On the other hand, the lack of a significant ipsi-contralateral modulation in the patients with UWS may depend on a loss of selective, top-down inhibition or, alternatively, subcortical facilitation of the Aβ-afferents from the hand, which selectively make synapse with the brainstem circuits subserving the HBR. In fact, an overall facilitation of HBR responses was appreciable, given that the HBR was recorded in all the patients^37,48,49^. Even though a systematic difference between ipsilateral and contralateral absolute AUC magnitude was appreciable, this difference failed to be significant when considering the whole pattern of AUC modulation within UWS and between the DoC groups. In fact, we focused our reasoning on the side-wise, whole AUC modulation instead of the stand-alone single sides.

### PPS tracing as a sign of awareness

Even though our findings help in differentiating patients with MCS (having top-down modulation) from those with UWS (lack of modulation) at both group and individual levels, the putative link between this finding (top-down modulation) and awareness (in terms of the level of behavioural responsiveness) is twofold.

First, the HBR magnitude (AUC) modulation was significantly correlated with the diagnosis based on the interpretation of the CRS-R scores. In fact, the logistic regression analysis showed that the correspondence between the diagnosis based on the interpretation of the CRS-R scores and the AUC modulation of HBR by hand position allowed to accurately predict the diagnosis (categorical variable) from the HBR modulation (continuous variable). Indeed, all patients with MCS showed an HBR modulation analogue to that shown by the HC (i.e., AUC^near^ > AUC^far^ > AUC^ultrafar^), whereas all subjects with UWS had a tonic, non-modulated HBR response (i.e., AUC^near^≃AUC^far^≃AUC^ultrafar^). Given that the CRS-R reflects the level of detrimental cortico-thalamo-cortical connectivity subserving awareness^98,99^, HBR might indeed represent an additional measure to suppose awareness preservation in patients with DoC, who should thus be further investigated with other instrumental approaches.

Second, it has been proposed that awareness generation and maintenance are mediated by vast cortico-thalamo-cortical networks encompassing frontal and parietal areas. The same areas support the PPS integrative functions, as demonstrated by animal and human studies documenting the role of the frontal cortex (post-arcuate premotor cortex), the right hemisphere (including the frontal and parietal cortex), the intraparietal sulcus, the lateral occipital complex, the premotor cortex, and the superior parietal occipital junction as critical regions for representing the visual space near the hand and the face^7,41,45,54^. Therefore, demonstrating such network preservation may be a potential indicator of awareness preservation.

### Limitations and conclusions

The present study has three main limitations. First, we assumed that the top-modulation of HBR may be a reflection of conscious awareness. However, it is known that the most of top-down modulations are below the level of consciousness. Further, saliency detection and attention do not necessarily require consciousness and vice-versa^100,101^, and the underlying networks are separated from the executive ones^102^. Thus, the reduced HBR modulations in patients with UWS could simply reflect a greater damage to central sensorimotor pathways (without any implication for consciousness).

Second, the sample enrolled was small. Consequently, further studies are necessary to confirm our findings, which indeed demonstrated a clear difference between MCS and UWS, also at individual level, as indicated by the LR data.

Third, we did not analyze the functional heterogeneity of the HBR as previously done^37,48,49^. This issue refers to the timely variations of the HBR feature within the recording window. Indeed, it has been reported that the effect of hand position was stronger in the second part of the HBR, whereas the effect of the recording side was exclusively present in the first part of the response^37,48,49^. These findings suggest that the HBR is not a unitary physiological phenomenon, but it is mediated functionally distinct components undergoing differential modulation. Thus, a deeper knowledge of the functional heterogeneity of the HBR, in DOC patients should be investigated, given that it could bring other new insight into DOC pathophysiology.

One could have doubts about a possible biasing effect of habituation phenomena on the consistency and repeatability of HBR. However, this issue has been ruled out formerly with the stimulation parameter employed^37,48,49^. The morphology of HBR was not abnormal in both the MCS and UWS. This is not surprising since brainstem structures and functions are usually preserved in patients with DoC^103^. In addition, the short distance between the stimulated hand and the face may prevent the potential effects of a deteriorated synchronization of afferent volleys along sensory pathways^104^.

## Conclusions

HBR responses are modulated by the hand-to-face distance in patients with MCS but not in those with UWS, in relation to their diagnosis based on the interpretation of the CRS-R scores. The grained modulation of a seemingly stereotyped defensive reflex response (i.e., the HBR) suggests residual preservation of the tonic and selective top-down projections from the cortical networks involved in PPS-related functions. Although it is possible that our findings will not be replicated in all patients with DoC, our approach promisingly contributes to the growing body of protocols aimed at refining the differential diagnosis between MCS and UWS. We could propose HBR assessment as a rapid and very easy tool to potentially differentiate between single individuals with MCS and UWS by identifying residual top-down modulation processes from higher-order cortical areas to sensory-motor integration networks related to the PPS.

## Data Availability

The datasets used and/or analysed during the current study available from the corresponding author on reasonable request.
